# Second law analysis with effects of Arrhenius activation energy and binary chemical reaction on nanofluid flow

**DOI:** 10.1038/s41598-020-57802-4

**Published:** 2020-01-27

**Authors:** Noor Saeed Khan, Poom Kumam, Phatiphat Thounthong

**Affiliations:** 1 Department of Mathematics, Abdul Wali Khan University, Mardan, 23200 Khyber Pakhtunkhwa Pakistan; 2 KMUTTFixed Point Research Laboratory, Room SCL 802 Fixed Point Laboratory, Science Laboratory Building, Department of Mathematics, Faculty of Science, King Mongkut’s University of Technology Thonburi (KMUTT), Bangkok, 10140 Thailand; 3 KMUTT-Fixed Point Theory and Applications Research Group, Theoretical and Computational Science Center (TaCS), Science Laboratory Building, Faculty of Science, King Mongkut’s University of Technology Thonburi (KMUTT), Bangkok, 10140 Thailand; 4 Department of Medical Research, China Medical University Hospital, China Medical University, Taichung, 40402 Taiwan; 5 Renewable Energy Research Center, Department of Teacher Training in Electrical Engineering, Faculty of Technical Education, King Mongkut’s University of Technology North Bangkok, 1518, Wongsawang, Bangsue, Bangkok, 10800 Thailand

**Keywords:** Mathematics and computing, Applied mathematics

## Abstract

The Arrhenius activation energy and binary chemical reaction are taken into account to consider the magnetohydrodynamic mixed convection second grade nanofluid flow through a porous medium in the presence of thermal radiation, heat absorption/generation, buoyancy effects and entropy generation. The items composing of the governing systems are degenerated to nonlinear ordinary differential equations by adopting the appropriate similarity transformations which are computed through Runge-Kutta-Fehlberg (RKF) numerical technique along with Shooting method. The solution is manifested through graphs which provides a detailed explanations of each profile in terms of involved parameters effects. The compared results maintain outstanding approach to the previous papers.

## Introduction

Porous medium is a substance retaining the stiff medium which is bound through holes. That stiff medium may have different structures or deformations. It is very easily understandable that the overall role of the pores construct the aid for multiphase flow. It is interestingly quite informative that during single phase flow, the acting pores carry the fluid and the same pores face the void area. The dispersion thermodynamics in permeable region have applications including mineral receiving, cloth preparation, keeping the extra material of rays emission in nuclear plant, etc. Considering the wide applications of porous media, Bhatti *et al*.^1^ showed the effects of coagulation (clotting of blood) in peristaltic type generated movement of an electrical nature possessing Prandtl liquid of physiological behavior in a tiny annular way having sinusoidal waves of peristaltic type proceeding with the inward and outward walls considerations at the same magnitude of velocity through a non-uniform annulus containing a homogeneous porous medium. Daniel *et al*.^2^ studied the time non-reliant current processing hydromagnetic movement and heating delivery generated due to tiny particles dispersion on a medium having pores of an expanding space using Buongiorno nanofluid model along with solar emission of rays, chemical reaction, heat emanating or converging, viscous and Ohmic dissipations. Investigating porous medium, Khan *et al*.^3^ treated the movement in heating prevailing system of a differential type dispersion on an expanding medium using series solution. Bhatti *et al*.^4^ presented the peristaltic study of heating and saturation transportation of two phase suspension movement involving chemistry properties via Darcy-Brinkman-Forchheimer space having pores carrying compliant boundaries for a particular type of suspension. Daniel^5^ took interest in determining the impact of motion bearing slip and inhaling effect on the wall for the time considering smooth heating layer motion on a plane space with heating conditions using analytical solution through homotopy analysis method. Khan *et al*.^6^ tested the thermal disorder, heat and mass transfer tiny dispersion movement with gyrotactic microorganisms in porous medium using heating wall information. Fetecau *et al*.^7^ investigated the flow without non-dimensional form, tangential force agents and the surface arising force relevant to the flow on account of plate existing in motion to deliver unique fascinating outcomes of the second issue presented by Stokes. Daniel *et al*.^8^ showed the effects of slipping, convective boundary conditions, solar emission of rays, viscous dissipation for two directional current processing hydromagnetic tiny dispersion movement past a porous nonlinear expanding/minimizing surface. Studies related to heat transfer and porous media can be seen in the references^9–19^.

In 1889, a Swedish scientist named Svante Arrhenius used the terminology activation energy for the first time. Activation energy denoted by *E*_*a*_ measured in KJ/mol represents the minimum energy attained through the atoms or molecules to initiate the chemical reaction. The quantity of activation energy is different for different chemical reactions, even some times it is zero. Activation energy (AE) with binary chemical reactions (BCR) exist in heat and mass transfer and have its applications in chemical engineering, geothermal reservoirs, emulsions of various suspensions, food processing *etc*. The first work on activation energy with binary chemical reaction was from Bestman^20^. Then other researchers like Hsiao^21^ composed a study of that topic for rich viscous fluid which undergoes the current in the prevailing environment of magnetohydrodynamics with some other factors on extrusion system to promote the system’s economic efficiency. Khan *et al*.^22^ paid attention to AE with BCR and entropy generation in Casson nanofluid enhancing consumption of reactive species with chemical parameter. Mustafa *et al*.^23^ analyzed AE with BCR in mixed convective movement of magneto-tiny-particles dispersion on an expandable space incorporating zero flux at the boundary in which the heating transfusion on behalf of the wall decreased on incrementing the CR rate quantity. Khan *et al*.^24^ focused their investigations on the AE with BCR in mixed convective MHD movement considering point of stagnation towards a stretching material accompanying solar rays emission and heating converging to a point or from a point which investigated the constituents saturation increased to the incremental magnitude in AE with BCR. Irfan *et al*.^25^ scrutinized the AE with BCR in nonlinear mixed convection unsteady Carreau tiny particles dispersion movement on a bidirectional stretching sheet in which the activation energy and thermophoresis were enhanced. Anuradha and Yegammai^26^ have presented the AE with BCR with the effects of loss due to rich thick fluid and Ohm notion on time involving two directional solar rays emission hydromagnetic two part movement of rich thick lacking incompressibility in current process tiny particles dispersion showing that rate of change of displacement and heating increased with the heat generation/absorption parameter. Maleque^27^ investigated activation energy accompanying both type of reactions of heating absorbing and evolving in the movement and heating transportation for which numerical solution was obtained through RKF with the collaboration of other procedure along with NS iteration procedure. Since activation energy is related to heating and saturation transferring so the heating and saturation transferring studies may be consulted in the references^28–45^.

Entropy generation is strongly dependent on system flow, heat and mass transfer. The outstanding work related to entropy generation is from Bejan^46^ conducted for the first time. Ellahi *et al*.^47^ opted for the disorderness in peristaltic type motion of tiny particles dispersion in a asymmetric way lying at right angle by discussing the prominent dominance of a heating conduction formulated in random scattering of tiny particles for tiny particles dispersions involving the projection of tiny particle body and particle saturation. Daniel *et al*.^48^ tackled down the issue of thermal disorderness in time non-reliant heating motion of a current passing tiny particles dispersion and heating transportation in a pores keeping linear expanding material accompanying the collective projections of electricity and MHD reliant fields, solar rays emission, viscousness loss, and species combination using implicit finite difference method in which the joint heating phenomena and parameters like buoyancy have reverse effects on Bejan number. Ishaq *et al*.^49^ worked on irreversibility in two directional tiny particle dispersion movement of Powell Eyring suspension accompanying heating and saturation transmitting on an expanding material keeping pores prevailing the similar magnitude of external agent proving that thermal disorderness kept incremental position on incrementing dissipative representative, Hartmann and other numbers. Daniel *et al*.^50^ presented the irreversibility phenomena and its reciprocal in time non-reliant heating motion of current passing hydromagnetic tiny particles dispersion with suction/injection at the wall using feasible and realistic applicable tiny particles dispersion formulation for assisting informations. Their achievements showed that entropy generation increased with the current passing environment, solar rays emission, and inhaling but decreased with the tiny particles zigzag behavior and external applied agent parameter. Khan *et al*.^51^ documented entropy generation for Sisko nanomaterial flow due to rotating stretchable disks investigating that entropy generation increased for increasing Brinkman number and diffusion.

Due to wide interest in energy sector, it is hoped that the present manuscript will explore a new area of research namely second law analysis with the projections of Arrhenius AE with BC on nanofluid movement through a Runge-Kutta-Fehlberg (RKF) numerical technique along collaboration of other procedure. The projections of numerous representatives on movement, heat transfer, concentration and entropy generation are revealed in graphs and debated.

## Problem Formulation

### Method

**Figure 1:**
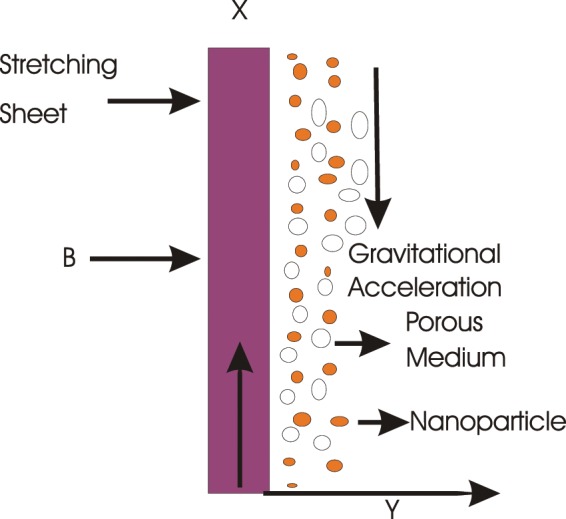
Problem geometry.

Steady two-dimensional hydromagnetic mixed convection flow of a second-grade nanofluid suspended with nanoparticles controlled through stretching sheet is scrutinized. Heat transfer carries the thermal radiation, Brownian motion, thermophoresis, heat source/sink, Joule heating and viscous dissipation. Binary chemical reaction and Arrhenius activation energy are incorporated. Magnetic field ***B*** = [0, *B*_0_, 0] is directed in *y*-direction. The significance of electric and magnetic fields are considered negligible on account of magnetic Reynolds number consideration to vanishing. Due to gravity, the gravitational acceleration is ***g*** = [0, *g*, 0]. Coordinate system is engaged in a manner that *x*-axis is directed in stretching side and *y*-axis lies normal to the stretching sheet (please note Fig. 1).

The governing equations are as in^22–27^

1 $$\frac{\partial u}{\partial x}+\frac{\partial v}{\partial y}=0,$$

2 $$\begin{array}{lll}{\rho }_{f}\left(u\frac{\partial u}{\partial x}+v\frac{\partial u}{\partial y}\right) & = & {u}_{e}\frac{d{u}_{e}}{dx}+{\mu }_{f}\frac{{\partial }^{2}u}{{\partial y}^{2}}+{\alpha }_{1}\left[\frac{\partial }{\partial x}\left(u\frac{{\partial }^{2}u}{{\partial y}^{2}}\right)-\frac{\partial u}{\partial y}\frac{{\partial }^{2}u}{\partial x\partial y}+v\frac{{\partial }^{3}u}{{\partial y}^{3}}\right]+\left[g{\beta }_{T}(T-{T}_{\infty })\right]\\  &  & -\left[g{\beta }_{C}(C-{C}_{\infty })\right]-\sigma {B}_{0}^{2}({u}_{e}-u)-\frac{{\nu }_{f}}{k}u,\end{array}$$

3 $$\begin{array}{lll}u\frac{\partial T}{\partial x}+v\frac{\partial T}{\partial y} & = & \lambda \frac{{\partial }^{2}T}{{\partial y}^{2}}+\tau \left[{D}_{B}\left(\frac{\partial C}{\partial y}\frac{\partial T}{\partial y}\right)+\left(\frac{{D}_{T}}{{T}_{\infty }}\right){\left(\frac{\partial T}{\partial y}\right)}^{2}\right]\\  &  & -\frac{1}{{(\rho {c}_{P})}_{f}}\left[\frac{{\partial q}_{r}}{\partial y}+q(T-{T}_{\infty })+{\alpha }_{1}\frac{\partial u}{\partial y}\left[\frac{\partial }{\partial y}\left(u\frac{\partial u}{\partial x}+v\frac{\partial u}{\partial y}\right)\right]+\sigma {B}_{0}^{2}{u}^{2}\right],\end{array}$$

4 $$u\frac{\partial C}{\partial x}+v\frac{\partial C}{\partial y}=\frac{{D}_{B}{\partial }^{2}C}{{\partial y}^{2}}+\frac{{D}_{T}}{{T}_{\infty }}\frac{{\partial }^{2}T}{{\partial y}^{2}}-{k}_{r}^{2}(C-{C}_{\infty }){\left[\frac{T}{{T}_{\infty }}\right]}^{m}\exp \left[\frac{-{E}_{a}}{\kappa T}\right],$$

where, *u*, *v* are the velocity components along *x* and *y*-axes and *u*_*w*_ is the stretching velocity. The subscripts f and *P* denote respectively the base fluid and pressure. *μ*_*f*_ is the dynamic viscosity, *σ* is the electrical conductivity and *ρ*_*f*_ is the density of the nanoliquid. *ν*_*f*_ = $$\frac{{\mu }_{f}}{{\rho }_{f}}$$ is the kinematic viscosity, *k* is the permeability of porous medium, *α*_1_( > 0) is the material parameter, *β*_*T*_ and *β*_*C*_ are respectively the thermal and concentration expansions, *T* and *C* are respectively the fluid temperature and concentration, *T*_*∞*_ and *C*_*∞*_ are respectively the fluid ambient temperature and concentration, *q*_*r*_ is the radiative heat flux, *q* is the heat source/sink parameter, *D*_*B*_ and *D*_*T*_ are respectively the Brownian and thermophoretic diffusion coefficients, *λ* = $$\frac{{k}_{1}}{{\rho }_{f}}$$ is the thermal diffusivity of the nanofluid in which *k*_1_ is the thermal conductivity, *k*_*r*_ is the rate (constant) of chemical reaction, *τ* = $$\frac{{(\rho c)}_{P}}{{(\rho c)}_{f}}$$ is the ratio of nanoparticles heat capacity and base fluid heat capacity. *m* is the fitted rate constant such that (−1 <  *m*  <  1), *E*_*a*_ is the activation energy, *κ* = 8.61  ×  10^−5^eV/K is the Boltzmann constant and $${k}_{r}^{2}$$(C – C_*∞*_)$${\left[\frac{T}{{T}_{\infty }}\right]}^{m}$$$$\exp $$$$\frac{-{E}_{a}}{\kappa T}$$ is the modified Arrhenius term.

The following boundary conditions are used

5 $$u={u}_{w}={c}_{1}x,\ \ v=0,\ \ T={T}_{w},\ \ C={C}_{w}\ \ at\ \ y=0,$$

6 $$u={u}_{e}={c}_{2}x,\ \ \frac{\partial u}{\partial y}\to 0,\ \ T\to {T}_{\infty },\ C\to {C}_{\infty }\ at\ \ y=\infty ,$$

where *c*_1_ and *c*_2_ are constants such that *c*_1_ >  0. Using Rosseland approximation^25^ for radiation term as

7 $${q}_{r}=-\frac{4{\sigma }_{1}}{3{k}_{2}}\frac{{\partial T}^{4}}{\partial y},$$

where *σ*_1_ and *k*_2_ are the Stefan-Boltzmann and the mean absorption coefficient respectively. Expanding *T*^4^ by Taylor’s series at *T*_*∞*_ and neglecting higher order terms

8 $${T}^{4}\ \approxeq \ 4{T}_{\infty }^{3}T-3{T}_{\infty }^{4}.$$

So

9 $$\frac{{\partial q}_{r}}{\partial y}=-\frac{16{T}_{\infty }^{3}{\sigma }_{1}}{3{k}_{2}}\frac{{\partial }^{2}T}{{\partial y}^{2}}.$$

The transformations used here are

10 $$\begin{array}{lll}\psi (x,y) & = & x{({c}_{1}{\nu }_{f})}^{\frac{1}{2}}f(\zeta ),\ u=\frac{\partial \psi }{\partial y}={c}_{1}x{f}^{^{\prime} }(\zeta ),\ v=\frac{\partial \psi }{\partial x}=-{({c}_{1}{\nu }_{f})}^{\frac{1}{2}}f(\zeta ),\ \zeta ={\left[\frac{{c}_{1}}{{\nu }_{f}}\right]}^{\frac{1}{2}}y,\\ \theta (\zeta ) & = & \frac{T-{T}_{\infty }}{{T}_{w}-{T}_{\infty }},\ \phi (\zeta )=\frac{C-{C}_{\infty }}{{C}_{w}-{C}_{\infty }},\end{array}$$

where *ψ* is the stream function. *f*, *ζ*, *θ* and *ϕ* are the dimensionless velocity, variable, temperature and concentration respectively. *T*_*w*_ and *C*_*w*_ are respectively the nanofluid temperature and concentration at the wall.

Continuity Eq. (1) is identically satisfied through Eq. (10). Using Eq. (10), the following five ordinary differential equations are formed from Eqs. (2)–(6)

11 $$f^{\prime\prime\prime} +ff^{\prime\prime} +\alpha \left(2f^{\prime} f^{\prime\prime\prime} -{f}^{^{\prime\prime} 2}-f{f}^{iv}\right)-{f^{\prime} }^{2}+{A}^{2}+M(A-f^{\prime} )+{\lambda }_{1}\theta +{\lambda }_{2}\phi -{\lambda }_{3}f^{\prime} =0,$$

12 $$\frac{1}{Pr}(\theta ^{\prime\prime} +Rd)+Nt\theta ^{\prime} \phi ^{\prime} +Nb({\theta ^{\prime} }^{2})+\gamma \theta +f\theta ^{\prime} +MEc{f}^{^{\prime} 2}+Ec{f}^{^{\prime\prime} 2}+\alpha f^{\prime\prime} \left(2f^{\prime} f^{\prime\prime} -ff^{\prime\prime\prime} \right)=0,$$

13 $$\frac{1}{Sc}{\phi }^{^{\prime\prime} }+f\phi ^{\prime} +\frac{1}{Sc}\frac{Nt}{Nb}{\theta }^{^{\prime\prime} }+{\gamma }_{1}{({\gamma }_{2}\theta +1)}^{m}\phi \exp \left(\frac{-E}{{\gamma }_{2}\theta +1}\right)=0,$$

14 $$f=0,\ f^{\prime} =1,\ \theta =1,\ \phi =1,\ at\ \zeta =0,$$

15 $$f^{\prime} =A,\ {f}^{^{\prime\prime} }=0,\ \theta =0,\ \phi =0\ at\ \zeta =\infty ,$$

where (′) is the differentiation with respect to *ζ*. *α* = $\frac{{c}_{1}{\alpha }_{1}}{{\mu }_{f}}$, $A=\frac{{c}_{2}}{{c}_{1}}$ and $M=\frac{\sigma {B}_{0}^{2}}{{c}_{1}{\rho }_{f}}$ are the non-dimensional second-grade nanofluid, rate constants ratio and magnetic field parameters respectively. *λ*_1_ = $\frac{Gr}{R{e}_{x}^{2}}$ and *λ*_2_ = $\frac{Gs}{R{e}_{x}^{2}}$ stand for the thermal and concentration buoyancy parameters in which $Gr=\frac{g{\beta }_{T}({T}_{w}-{T}_{\infty }){x}^{3}}{{\nu }_{f}^{2}}$ and $Gs=\frac{g{\beta }_{C}({C}_{w}-{C}_{\infty }){x}^{3}}{{\nu }_{f}^{2}}$ are the thermal and solutal Grashof numbers, where $R{e}_{x}=\frac{x{u}_{w}}{{\nu }_{f}}$ is the local Reynolds number. ${\lambda }_{3}=\frac{{\nu }_{f}}{k{c}_{1}}$, $Rd=\frac{16{T}_{\infty }^{3}{\sigma }_{1}}{3{k}_{2}\lambda },Nt=\tau \frac{{D}_{T}({T}_{w}-{T}_{\infty })}{{T}_{\infty }{\nu }_{f}}$, $Nb=\tau \frac{{D}_{B}({C}_{w}-{C}_{\infty })}{{\nu }_{f}}$, *γ* = $\frac{q}{{c}_{1}{(\rho {c}_{P})}_{f}}$, *γ*_1_ = $\frac{{k}_{r}^{2}}{{c}_{1}}$, *γ*_2_ = $\frac{{T}_{w}-{T}_{\infty }}{{T}_{\infty }}$ and $E=\frac{{E}_{a}}{\kappa {T}_{\infty }}$ are the porosity, thermal radiation, thermophoresis, Brownian motion, heat source/sink, chemical reaction, temperature difference and non-dimensional activation energy parameters respectively. Similarly $Pr=\frac{{\nu }_{f}}{\lambda }$, $Ec=\frac{{c}_{1}^{2}{x}^{2}}{({T}_{w}-{T}_{\infty })}$ and $Sc=\frac{{\nu }_{f}}{{D}_{B}}$ are the Prandtl, Eckert and Schmidt numbers respectively.

For *α* = 0 and *M* = 0, the study is about viscous nanofluid in the absence of magnetic field.

The physical quantities of practical interests are the local skin friction coefficient ${C}_{{f}_{x}}$, the local Nusselt number *Nu*_*x*_ and the local wall mass transfer rate *Sh*_*x*_ having the descriptions

16 $${C}_{{f}_{x}}=\frac{{\tau }_{w}}{{\rho }_{f}{u}_{w}^{2}},\ \ N{u}_{x}=\frac{{q}_{w}x}{{k}_{1}({T}_{w}-{T}_{\infty })},\ \ S{h}_{x}=\frac{{q}_{m}x}{{D}_{B}({C}_{w}-{C}_{\infty })},$$

where

17 $$\begin{array}{lll}{\tau }_{w} & = & {\left[{\mu }_{f}\frac{\partial u}{\partial y}+{\alpha }_{1}\left(u\frac{{\partial }^{2}u}{\partial x\partial y}+v\frac{{\partial }^{2}u}{{\partial y}^{2}}-2\frac{\partial u}{\partial x}\frac{\partial v}{\partial y}\right)\right]}_{y=0},\\ {q}_{w} & = & -{k}_{1}{\left(\frac{\partial T}{\partial y}\right)}_{y=0}-\frac{4{\sigma }_{1}}{3{k}_{2}}{\left[\frac{{\partial T}^{4}}{\partial y}\right]}_{y=0},\\ {q}_{m} & = & -{D}_{B}{\left(\frac{\partial C}{\partial y}\right)}_{y=0}.\end{array}$$

Here *τ*_*w*_, *q*_*w*_ and *q*_*m*_ are the shear stress, heat flux and mass flux respectively at the surface.

Substituting the required values from Eq. (17) in Eq. (16) and using Eq. (10), one obtains

18 $${C}_{{f}_{x}}={(R{e}_{x})}^{-\frac{1}{2}}\left[1+3\alpha \right]{f}^{^{\prime\prime} }(0),\ N{u}_{x}=-{(R{e}_{x})}^{\frac{1}{2}}(1+Rd)\theta ^{\prime} (0),\ S{h}_{x}=-{(R{e}_{x})}^{\frac{1}{2}}\phi ^{\prime} (0).$$

## Entropy Generation

Entropy generation is given as

19 $$\begin{array}{c}{E}_{gen}^{{\rm{^{\prime} }}{\rm{^{\prime} }}{\rm{^{\prime} }}}=\frac{{k}_{1}}{{T}_{{\rm{\infty }}}^{2}}\left[{\left(\frac{{\rm{\partial }}T}{{\rm{\partial }}y}\right)}^{2}+\frac{16{T}_{{\rm{\infty }}}^{3}{\sigma }_{1}}{3{k}_{2}}{\left(\frac{{\rm{\partial }}T}{{\rm{\partial }}y}\right)}^{2}\right]+\frac{{\mu }_{f}}{{T}_{{\rm{\infty }}}}{\left(\frac{{\rm{\partial }}u}{{\rm{\partial }}y}\right)}^{2}+\frac{{\mu }_{f}{\alpha }_{1}}{{T}_{{\rm{\infty }}}}\left(u\frac{{\rm{\partial }}u}{{\rm{\partial }}y}\frac{{{\rm{\partial }}}^{2}u}{{\rm{\partial }}x{\rm{\partial }}y}+v\frac{{\rm{\partial }}u}{{\rm{\partial }}y}\frac{{{\rm{\partial }}}^{2}u}{{\rm{\partial }}{y}^{2}}\right)+\frac{\sigma {B}_{0}^{2}}{{T}_{{\rm{\infty }}}}{u}^{2}+\frac{RD}{{C}_{{\rm{\infty }}}}{\left(\frac{{\rm{\partial }}C}{{\rm{\partial }}y}\right)}^{2}+\frac{RD}{{T}_{{\rm{\infty }}}}\left[\frac{{\rm{\partial }}T}{{\rm{\partial }}x}\frac{{\rm{\partial }}C}{{\rm{\partial }}x}+\frac{{\rm{\partial }}T}{{\rm{\partial }}y}\frac{{\rm{\partial }}C}{{\rm{\partial }}y}\right],\end{array}$$

where *R* and *D* are the ideal gas constant and diffusion respectively. In Eq. (19), the first, second, third, fourth, fifth and sixth terms are respectively irreversibilities due to heat transfer with thermal radiation, viscous dissipation, second grade nanofluid friction, magnetic field and diffusion effects. The characteristic irreversibility (entropy generation) rate is

20 $${E}_{0}^{{\rm{^{\prime} }}{\rm{^{\prime} }}{\rm{^{\prime} }}}=\frac{{k}_{1}{({T}_{w}-{T}_{{\rm{\infty }}})}^{2}}{{x}^{2}{T}_{{\rm{\infty }}}^{2}}.$$

The non-dimensional entropy generation rate *N*_*G*_(*ζ*) is obtained through Eq. (10) using

21 $${N}_{G}(\zeta )=\frac{{E}_{gen}^{^{\prime\prime\prime}}}{{E}_{0}^{^{\prime\prime\prime}}}.$$

So

22 $$\begin{array}{lcc}{N}_{G}(\zeta ) & = & Re\left[1+Rd{(1+({\gamma }_{2}-1){\gamma }_{2})}^{3}\right]{(\theta ^{\prime} )}^{2}+\frac{ReBr}{{({\gamma }_{2}-1)}^{2}}\left[{(f^{\prime\prime} )}^{2}+M{(f^{\prime} )}^{2}\right]+\frac{ReBr\alpha }{{({\gamma }_{2}-1)}^{2}}\left[f{(f^{\prime\prime} )}^{2}-ff^{\prime} f^{\prime\prime\prime} \right]\\  &  & +\,Re{\gamma }_{3}\left(\frac{{\phi }_{w}}{{\gamma }_{2}}\right){(\phi ^{\prime} )}^{2}+\,Re{\gamma }_{3}\left(\frac{{\phi }_{w}}{{\gamma }_{2}}\right)\theta ^{\prime} \phi ^{\prime} ,\end{array}$$

where $Re=\frac{x{u}_{w}(x)}{{\nu }_{f}}$, $Br=\frac{{\mu }_{f}{u}_{w}^{2}}{{k}_{1}{T}_{{\rm{\infty }}}}$, *γ*_3_ = $\frac{RD{C}_{{\rm{\infty }}}}{{k}_{1}}$ and *ϕ*_*w*_ = $\frac{{C}_{w}-{C}_{\infty }}{{C}_{\infty }}$ are respectively Reynolds number, Brinkman number, diffusion parameter due to nanoparticles concentration and nanoparticles concentration difference parameter.

## Results and discussion

The non-dimensional Eqs. (11)–(15) have been computed through MATLAB built in routine *bvp4c*. Equations (18) and (22) are computed through the achieved solution of MATLAB built in routine *bvp4c*. The problem geometry is shown in Fig. 1. The effects of various parameters on velocity, temperature, concentration and entropy generation rate are shown in Fig. 2–25 respectively. There exists a close agreement in the results of present and published work in Table 1.

**Figure 2:**
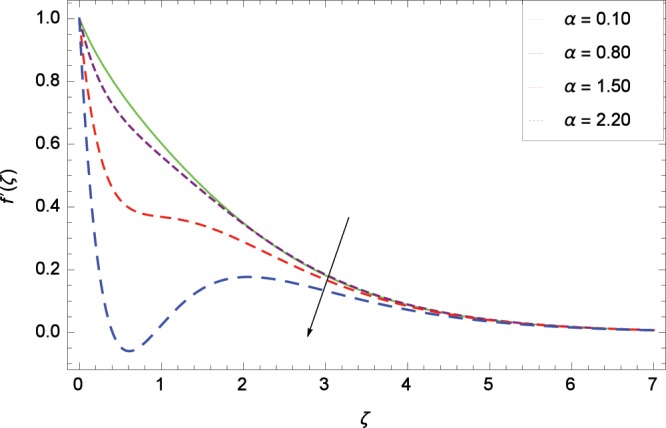
Influence of *α* on velocity $f^{\prime}$(*ζ*).

**Figure 3:**
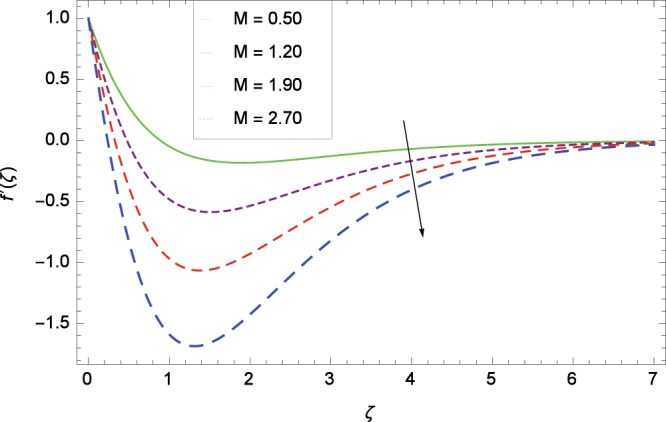
Influence of *M* on velocity $f^{\prime}$(*ζ*).

**Figure 4:**
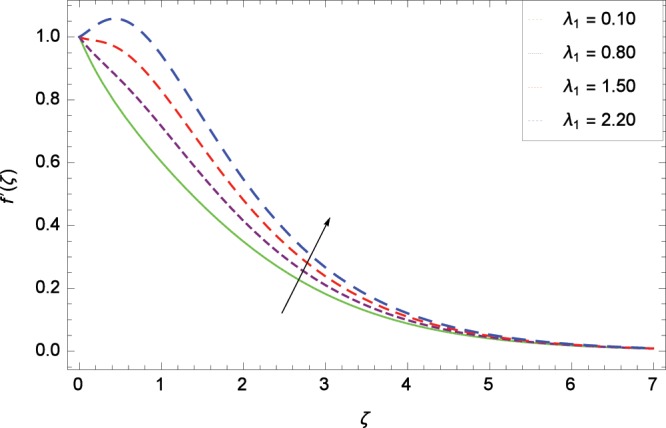
Influence of *λ*_1_ on velocity $f^{\prime}$(*ζ*).

**Figure 5:**
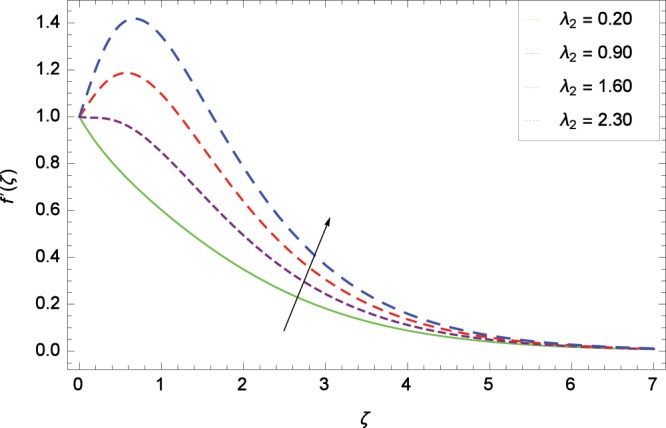
Influence of *λ*_2_ on velocity ${f}^{^{\prime} }$(*ζ*).

**Figure 6:**
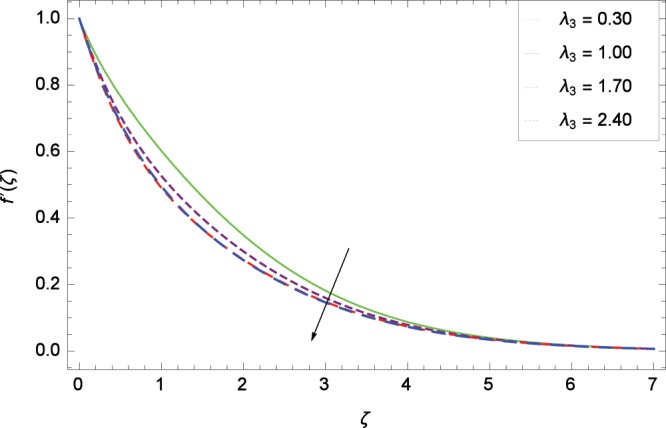
Influence of *λ*_3_ on velocity ${f}^{^{\prime} }$(*ζ*).

**Figure 7:**
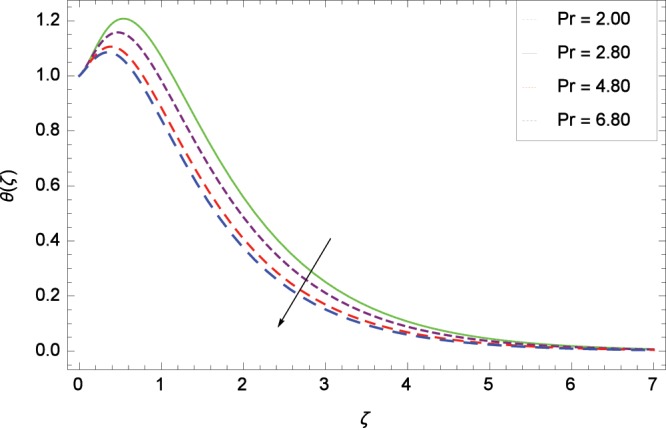
Influence of *Pr* on temperature *θ*(*ζ*).

**Figure 8:**
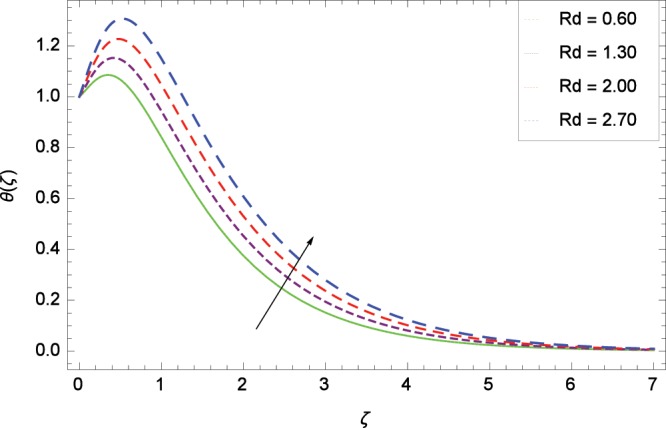
Influence of *Rd* on temperature *θ*(*ζ*).

**Figure 9:**
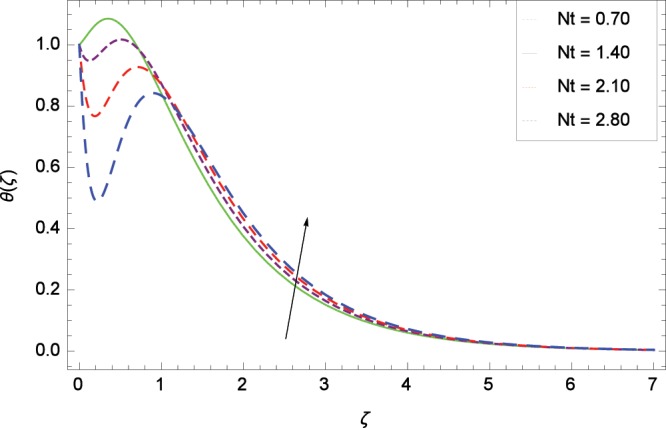
Influence of *Nt* on temperature *θ*(*ζ*).

**Figure 10:**
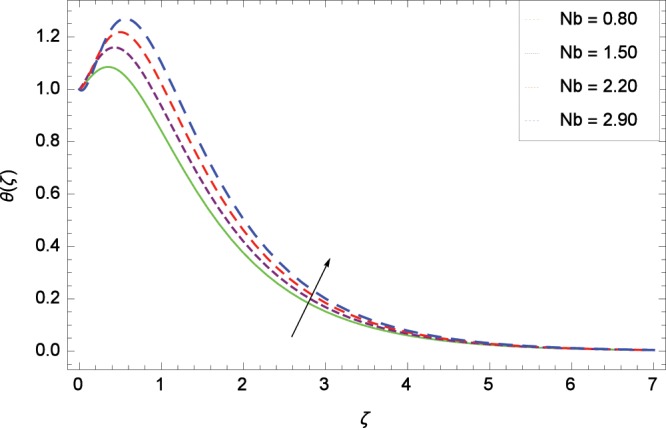
Influence of *Nb* on temperature *θ*(*ζ*).

**Figure 11:**
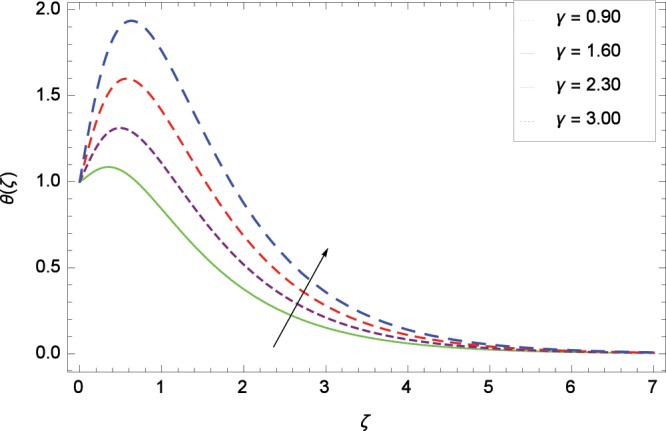
Influence of *γ* on temperature *θ*(*ζ*).

**Figure 12:**
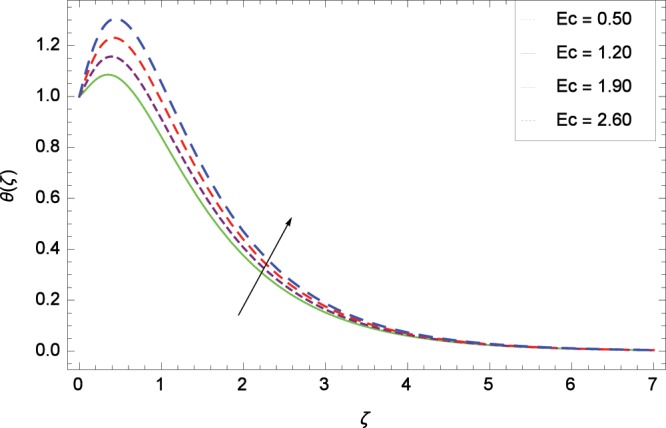
Influence of *Ec* on temperature *θ*(*ζ*).

**Figure 13:**
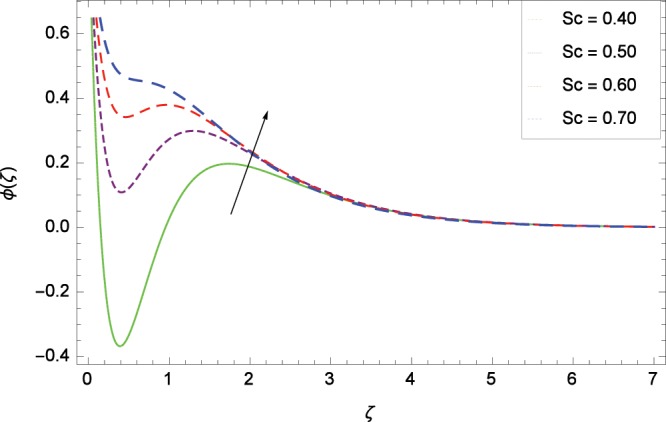
Influence of *Sc* on concentration *ϕ*(*ζ*).

**Figure 14:**
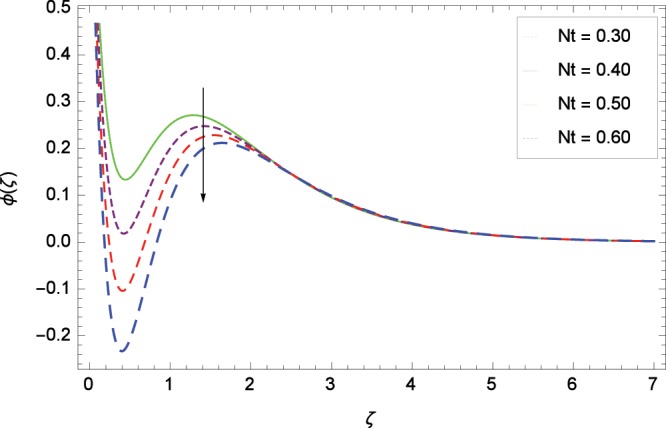
Influence of *Nt* on concentration *ϕ*(*ζ*).

**Figure 15:**
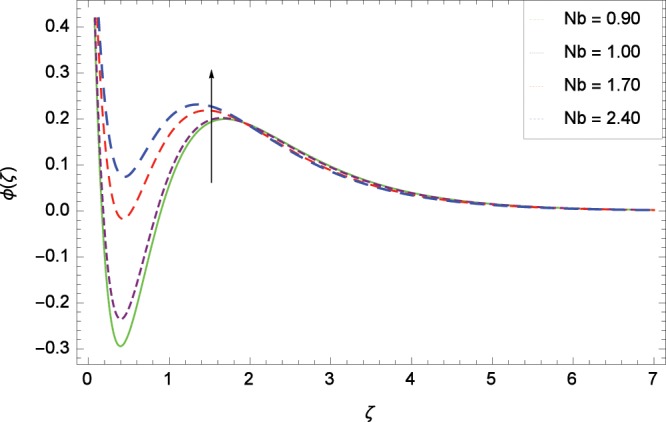
Influence of *Nb* on concentration *ϕ*(*ζ*).

**Figure 16:**
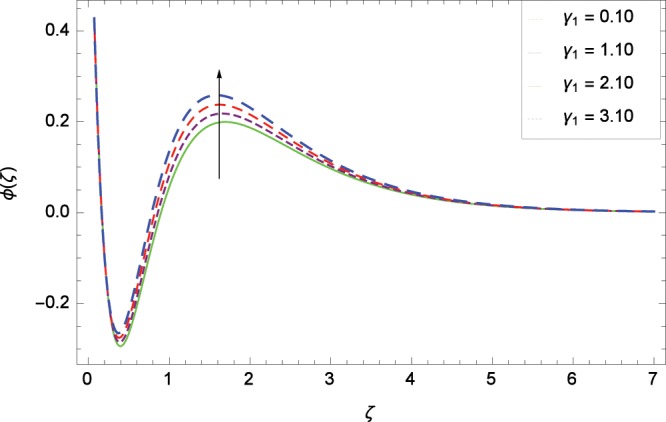
Influence of *γ*_1_ on concentration *ϕ*(*ζ*).

**Figure 17:**
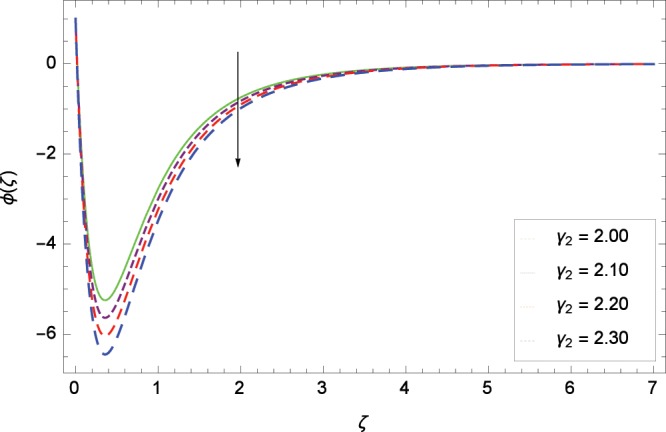
Influence of *γ*_2_ on concentration *ϕ*(*ζ*).

**Figure 18:**
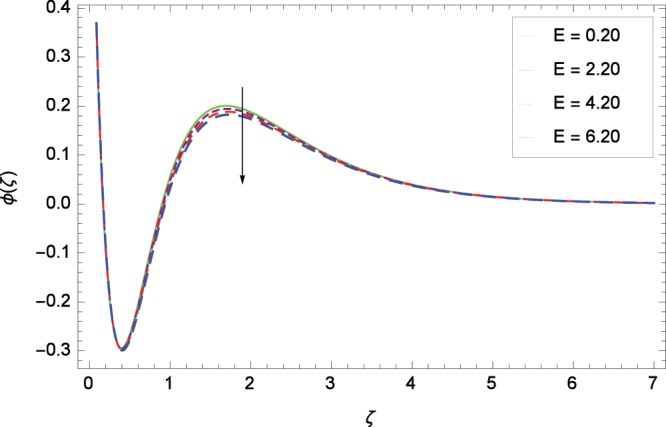
Influence of *E* on concentration *ϕ*(*ζ*).

**Figure 19:**
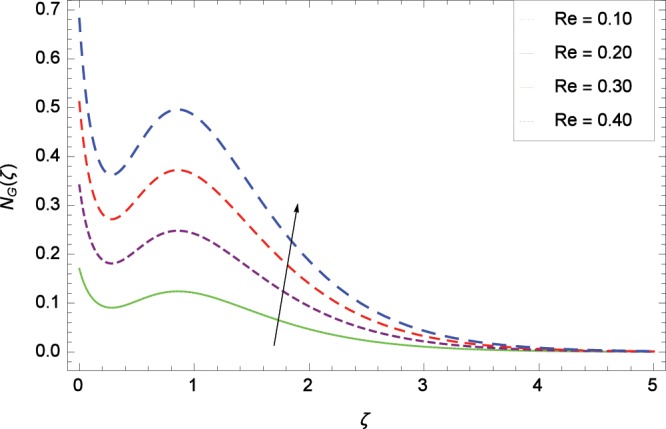
Influence of *Re* on entropy generation rate *N*_*G*_(*ζ*).

**Figure 20:**
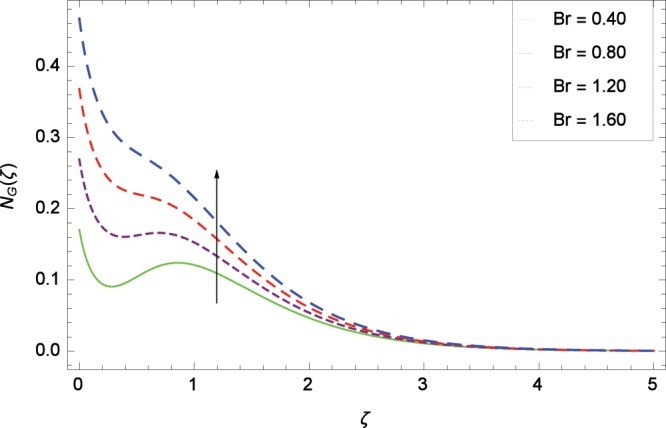
Influence of *Br* on entropy generation rate *N*_*G*_(*ζ*).

**Figure 21:**
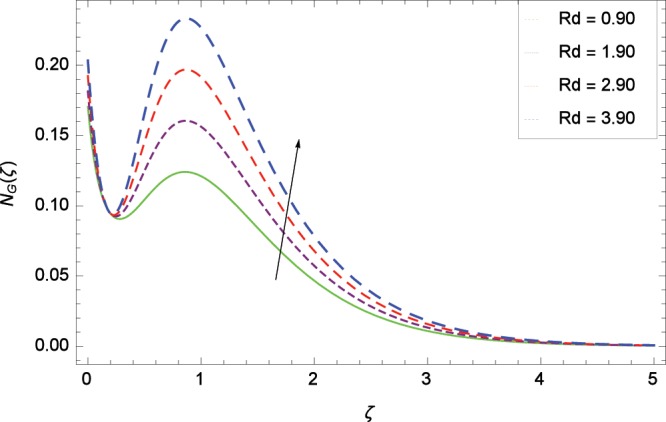
Influence of *Rd* on entropy generation rate *N*_*G*_(*ζ*).

**Figure 22:**
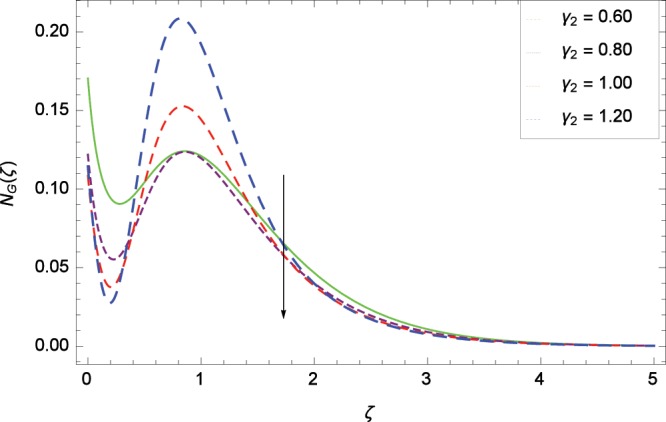
Influence of *γ*_2_ on entropy generation rate *N*_*G*_(*ζ*).

**Figure 23:**
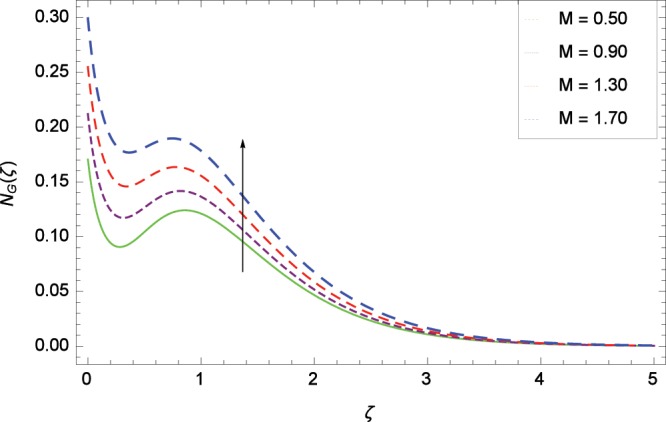
Influence of *M* on entropy generation rate *N*_*G*_(*ζ*).

**Figure 24:**
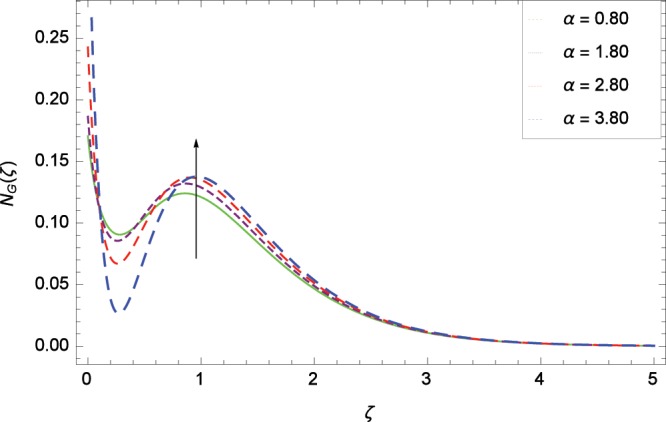
Influence of *α* on entropy generation rate *N*_*G*_(*ζ*).

**Figure 25:**
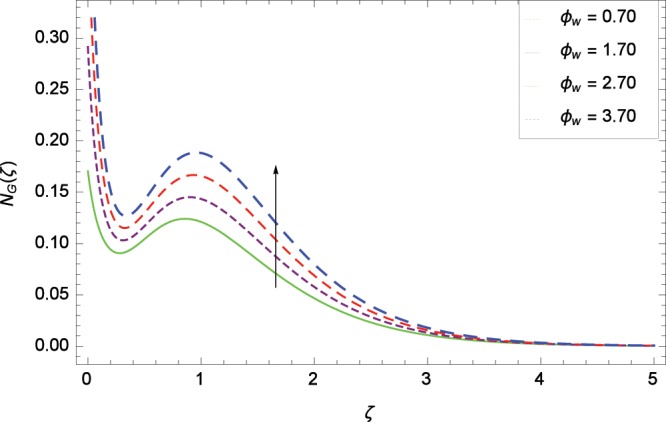
Influence of *ϕ*_*w*_ on entropy generation rate *N*_*G*_(*ζ*).

**Table 1:** Comparison of the present work with the published literature.

${C}_{{f}_{x}}$^24^	${C}_{{f}_{x}}$ (Present)	*Nu*_*x*_^24^	*Nu*_*x*_ (Present)	*Sh*_*x*_^24^	*Sh*_*x*_ (Present)
− 1.44442	− 1.44441	− 0.157986	− 0.157985	− 0.0700247	− 0.0700246
− 1.55686	− 1.55687	− 0.159360	− 0.159359	− 0.0729418	− 0.0729417
− 1.68828	− 1.68827	− 0.159951	− 0.159950	− 0.0752293	− 0.0752292
− 1.65462	− 1.65463	− 0.159204	− 0.159203	− 0.0770811	− 0.0770810
− 1.62147	− 1.62145	− 0.158443	− 0.158442	− 0.0427246	− 0.0427245
− 1.58882	− 1.58881	− 0.156645	− 0.156644	− 0.0282003	− 0.0282002
− 1.55664	− 1.55663	− 0.155620	− 0.155621	− 0.0152936	− 0.0152935
− 1.39845	− 1.39844	− 0.154592	− 0.154591	− 0.0621507	− 0.0621506
− 1.20846	− 1.20845	− 0.153259	− 0.153258	− 0.0721547	− 0.0721546
− 0.99442	− 0.99441	− 0.152927	− 0.152926	− 0.0765287	− 0.0765286
− 0.76381	− 0.76380	− 0.152573	− 0.152572	− 0.0789951	− 0.0789950
− 0.84731	− 0.84730	− 0.151476	− 0.151475	− 0.0784224	− 0.0784223
− 0.88528	− 0.88527	− 0.150990	− 0.150991	− 0.0779765	− 0.0779764

### Velocity profile

Non-Newtonian nanofluid effect decreases the velocity ${f}^{^{\prime} }$(*ζ*) on getting the rising values of *α*. It is observed in Fig. 2 that the increasing values of *α* increase the viscosity of fluid hence decrease the velocity. Magnetic field is causing a resistive type force known as Lorentz force so in the presence of transverse magnetic field, an electrically conducting second grade nanofluid provides resistance to the flow thereby velocity ${f}^{^{\prime} }$(*ζ*) decreases as shown in Fig. 3. The thermal buoyancy parameter *λ*_1_ is showing its effect in Fig. 4. The velocity profile is increased for the higher values of *λ*_1_ which shows that nanofluid flow behavior increases across the vertical surface due to prevailing strength of gravity. The concentration buoyancy parameter *λ*_2_ increases the velocity ${f}^{^{\prime} }$(*ζ*) as depicted in Fig. 5. *λ*_2_ is the ratio of the buoyancy force to the viscous momentum force. The second grade nanofluid velocity ${f}^{^{\prime} }$(*ζ*) increases distinctively due to an enhancement in the species viscous momentum force on vertical surface at the cost of gravity. Figure 6 demonstrates the effect of porosity parameter *λ*_3_ on velocity ${f}^{^{\prime} }$(*ζ*). Porosity is related to the permeability of porous medium. The permeability refers to the capability of a porous material to allow liquids to pass through it. So increasing the porosity parameter *λ*_3_ increases the pores consequently, the flow is decreased due to resistance of pores.

### Temperature profile

The effect of second-grade nanofluid indicates that nanofluid have better heat transfer characteristics than the base fluid. Figure 7 depicts the influence of Prandtl number on temperature. It is worth mentioning that increasing values of *Pr* decrease the temperature since the thermal boundary layer is made thin. Prandtl number is the ratio of momentum to thermal diffusivity. Therefore high values of Prandtl number lead to stronger momentum diffusivity and low thermal diffusivity. Figure 8 reveals that temperature *θ*(*ζ*) is increased to high quantity in the presence of thermal radiation parameter *Rd*. The thermal radiation intensity means a reduction in the absorption coefficient so thermal radiation plays a significant role in the surface heat transfer where the convection heat transfer coefficient is low. Figure 9 shows that temperature is enhanced on high values of thermophoresis parameter *Nt* enriching the heat transport properties of second grade nanofluid. In the mean time the temperature and thermal boundary layer thickness are made high. Since the thermophoretic force is affected by temperature gradient so the heated particles are dragged away from hot to cold surface hence the thermal conductivity is improved. The Brownian motion parameter *Nb* is directed to enhance the temperature *θ*(*ζ*) filling Fig. 10. Brownian motion parameter increases the boundary layer thickness since the Brownian motion causes micro-mixing which improves the thermal conductivity of the nanofluid. The succession values of heat source/sink parameter *γ* increase the temperature for positive values i. e. *γ* > 0 (considering heat source) through Fig. 11. *γ* < 0 represents the heat sink case and *γ* = 0 shows the absence of heat source/sink in the thermal portion. Figure 12 shows the effect of Eckert number *Ec* on temperature profile *θ*(*ζ*) dedicated to boost the temperature due to frictional heating.

### Concentration profile

 Figure 13 shows the effect of Schmidt number *Sc* on nanoparticles concentration *ϕ*(*ζ*). Since *Sc* is the ratio of kinematic viscosity to molecular diffusivity so when *Sc* is enhanced nanoparticles concentration is increased. In Fig. 14, the effect of thermophoresis parameter *Nt* on nanoparticles concentration *ϕ*(*ζ*) is shown. Higher values of thermophoresis parameter weaken the thermpophoretic force which lead to the flow of nanoparticles from the region connected to tendency of high thermal energy. In other words, the flow of nanoparticles from high energy region to low energy region is reduced. Figure 15 shows the influence of Brownian motion parameter *Nb* on nanoparticles concentration *ϕ*(*ζ*). The random motion of the nanoparticles in the fluid at micro-scale level results in increment in the concentration. The binary chemical reaction parameter *γ*_1_ and nanoparticles concentration *ϕ*(*ζ*) up-gradations are elucidated in Fig. 16. Concentration is evolved due to the same phase of chemical reaction, nanoparticles and fluid molecules reactants. The temperature difference parameter *γ*_2_ decreases the concentration profile *ϕ*(*ζ*) located in Fig. 17. The thickness of concentration field is decreased for increasing values of *γ*_2_. Figure 18 shows that concentration profile *ϕ*(*ζ*) is not high through the increasing values of activation energy parameter *E*. It is watched that there is no sign to promote the concentration for the modified Arrhenius function, consequently, the general chemical reaction is improved.

### Irreversibility (entropy generation rate)

The influence of Reynolds number *Re* on entropy generation rate *N*_*G*_(*ζ*) is depicted in Fig. 19. With increasing Reynolds number *Re*, the chaos flow of second grade nanofluid is improved. It is watched in Fig. 20 that entropy is increased for larger values of *Br*. The reason is that large amount of heat is produced in the thermal system which favors the irreversibility. Figure 21 is specified for entropy generation rate *N*_*G*_(*ζ*) and thermal radiation parameter *Rd*. High thermal radiation is associated with the excessive temperature which in turns increases disorderedness in the system. The only parameter which decreases the entropy generation rate *N*_*G*_(*ζ*) is the temperature difference parameter *γ*_2_ as depicted in Fig. 22 hence chaos is controllable through *γ*_2_. In Fig. 23, the entropy generation rate *N*_*G*_(*ζ*) increases on increasing the magnetic field parameter *M*. It is due to the fact that Lorentz forces due to magnetic field generate dragging which causes the extra irreversibility in the system. The non-Newtonian second grade nanofluid parameter *α* sufficiently increases the production of irreversibility *N*_*G*_(*ζ*) which is shown in Fig. 24. Due to non-Newtonian effect, the viscous forces are excited and generate the chaos further. The nanoparticles concentration difference parameter *ϕ*_*w*_ increases the irreversibility *N*_*G*_(*ζ*) as depicted in Fig. 25. Nanoparticles are bodies which improve the thermal conduction so very easily increase the entropy generation rate.

### Comparison of the physical quantities with published work

It is noticed that in the present investigations, the skin friction coefficient and Nusselt number are increased while the Sherwood number has no consistency for increasing and decreasing behaviors when the relevant parameters have variations in their values like^24^.

## Conclusions

A study is conducted about the Arrhenius activation energy with binary chemical reaction and entropy analysis in nanofluid flow inducting differential equations and their solution for the influences of active parameters. The findings are given below.

1. Velocity decreases with increasing the parameters of second grade nanofluid, thermal radiation, magnetic field, porosity and increases with the parameters of thermal and solutal buoyancy parameters.
2. Temperature decreases with increasing the Prandtl number and increases with the parameters of thermophoresis, Brownian motion, heat source/sink, and Eckert number.
3. Concentration decreases with increasing the parameters of thermophoresis, temperature difference, activation energy and increases with increasing the Schmidt number, Brownian motion and chemical reaction parameters.
4. Entropy generation decreases with temperature difference parameter and increases with increasing Reynolds number, Brownian motion, thermal radiation, magnetic field, second grade nanofluid, nanoparticle concentration difference parameters.
5. The excellent agreement in the results of present and published work has been shown through Table 1.

## Data Availability

All the relevant material is available.
